# Raw milk special collection

**DOI:** 10.1017/S0950268822000930

**Published:** 2022-06-15

**Authors:** Norman Noah

**Affiliations:** London School of Hygiene and Tropical Medicine, London, UK

Milk is usually associated with human kindness, with lands of honey, and with other things nice. And it's good nutritionally for you, too!

It is a different story when milk is unpasteurised or otherwise contaminated. Despite the benefits of pasteurisation which have been known for decades, outbreaks from milk and dairy products are not uncommon. Some are caused by the unpasteurised product, others by inadequate pasteurisation, or by contamination after pasteurisation. Illness from contaminated milk and its products can be serious, and can still cause death, as exemplified in this recent collection of papers from our authors.

With an increasing global trend towards more ‘healthy’ and ‘natural’ unprocessed foods, we may well expect more outbreaks of infection from unpasteurised milk. In one study in England, (Mc Lauchlin), 5% of all dairy products made from raw milk were found to be potentially hazardous, and those with unsatisfactory bacterial counts accounted for a further significant proportion. The organisms most commonly reported from drinking raw milk in this collection are campylobacter (Davys, Kenton, Jenkins) and *E. coli* (Jenkins). Both these organisms can cause illness at very low doses. Campylobacter infection from this source also occurred in England from birds pecking the foil tops of pasteurised milk bottles left on doorsteps; most milk now sold is in cartons. Extensive salmonella infections still occur (Robinson). There is also further evidence that even *Coxiella burnetii*, though fortunately not *Q* fever as far as we know, may be transmitted by raw milk (Miller). Raw milk from camels can be hazardous too, as can milk from goats (Roess, Robinson).

Accidents during the pasteurisation process can occur (Jenkins). Fortunately, advances in making raw milk safer are on the way, as illustrated in the paper by Berge, which describes practices that have been developed for safer raw milk production.

Powdered infant milk has also been known to cause extensive outbreaks. Currently there is a large outbreak in US caused by cronobacter [1], and another caused by contamination of the milk cartons with sanitiser [2].

Cheese made from unpasteurised milk is legal in many countries, and can be safe, because the cheese-making process generally kills most harmful organisms. Nevertheless sometimes organisms such as Salmonella (Robinson), *E. coli* and Listeria occasionally survive, leading to illness. The French Agency for Food, Environmental and Occupational Health and Safety (ANSES) identified the types of unpasteurised milk cheeses on which to target efforts. In France, over the past decade, 34%, 37% and 60% of outbreaks of salmonellosis, listeriosis and *E. coli* infections have been linked to raw milk cheeses (Food Safety News, newsdesk 20 April 2022) (https://www.foodsafetynews.com/2022/04/anses-identifies-main-hazards-in-raw-milk-cheeses-e-coli-infections-top-the-list/).

We at Epidemiology and Infection strongly recommend that cheeses made from unpasteurised milk should be more readily identifiable than they are at present, perhaps with an easily visible red dot on the packet.

Milk and its products are wonderful and important adjuncts to most human diets. They remain, on the whole, wholesome and relatively safe: we hope this collection of papers will help towards making them even safer.
